# Nanoparticles augment the therapeutic window of RT and immunotherapy for treating cancers: pivotal role of autophagy

**DOI:** 10.7150/thno.77233

**Published:** 2023-01-01

**Authors:** Yuan-Hua Wu, Rong-Jane Chen, Hui-Wen Chiu, Li-Xing Yang, Yung-Li Wang, Yu-Ying Chen, Ya-Ling Yeh, Mei-Yi Liao, Ying-Jan Wang

**Affiliations:** 1 Department of Radiation Oncology, National Cheng Kung University Hospital, College of Medicine, National Cheng Kung University, Tainan 704, Taiwan.; 2 Department of Food Safety/Hygiene and Risk Management, College of Medicine, National Cheng Kung University, Tainan 704, Taiwan.; 3 Graduate Institute of Clinical Medicine, College of Medicine, Taipei Medical University, Taipei 110, Taiwan.; 4 Department of Medical Research, Shuang Ho Hospital, Taipei Medical University, New Taipei City 234, Taiwan.; 5 TMU Research Center of Urology and Kidney, Taipei Medical University, Taipei 110, Taiwan.; 6 Institute of Oral Medicine and Department of Stomatology, College of Medicine, National Cheng Kung University Hospital, National Cheng Kung University, Tainan 701, Taiwan.; 7 Department of Environmental and Occupational Health, College of Medicine, National Cheng Kung University, Tainan 704, Taiwan.; 8 Department of Applied Chemistry, National Pingtung University, Pingtung 900, Taiwan.; 9 Department of Medical Research, China Medical University Hospital, China Medical University, Taichung 404, Taiwan.

**Keywords:** radiotherapy, immunotherapy, nanomaterials, autophagy, tumor microenvironment

## Abstract

Immunotherapies are now emerging as an efficient anticancer therapeutic strategy. Cancer immunotherapy utilizes the host's immune system to fight against cancer cells and has gained increasing interest due to its durable efficacy and low toxicity compared to traditional antitumor treatments, such as chemotherapy and radiotherapy (RT). Although the combination of RT and immunotherapy has drawn extensive attention in the clinical setting, the overall response rates are still low. Therefore, strategies for further improvement are urgently needed. Nanotechnology has been used in cancer immunotherapy and RT to target not only cancer cells but also the tumor microenvironment (TME), thereby helping to generate a long-term immune response. Nanomaterials can be an effective delivery system and a strong autophagy inducer, with the ability to elevate autophagy to very high levels. Interestingly, autophagy could play a critical role in optimal immune function, mediating cell-extrinsic homeostatic effects through the regulation of danger signaling in neoplastic cells under immunogenic chemotherapy and/or RT. In this review, we summarize the preclinical and clinical development of the combination of immunotherapy and RT in cancer therapy and highlight the latest progress in nanotechnology for augmenting the anticancer effects of immunotherapy and RT. The underlying mechanisms of nanomaterial-triggered autophagy in tumor cells and the TME are discussed in depth. Finally, we suggest the implications of these three strategies combined together to achieve the goal of maximizing the therapeutic advantages of cancer therapy and show recent advances in biomarkers for tumor response in the evaluation of those therapies.

## 1. Introduction

Immune checkpoints play an important role in maintaining homeostasis of the immune system. Increasing research has indicated that in many types of cancers, immune checkpoints are overactivated, resulting in escape of immune surveillance on tumor cells 1. Therefore, immune checkpoint inhibitors (ICIs), including cytotoxic T lymphocyte antigen-4 (CTLA-4), programmed cell death protein 1 (PD-1)/programmed cell death-ligand 1 (PD-L1) axis, indoleamine 2,3-dioxygenase (IDO), etc., are now the frontline of immunotherapy for a variety of cancers 2. Accordingly, cancer immunotherapy that utilizes the host's immune system to fight against cancer cells has gained increasing interest in clinical trials due to its durable efficacy and low toxicity compared to traditional antitumor treatments, such as chemotherapy and radiotherapy (RT). Although ICIs have been investigated for more than two decades, many unanswered questions about their application in antitumor treatment remain to be solved 1. In particular, how to achieve durable responses to metastatic or inoperable malignancies and minimize the side effects of ICIs need to be resolved. The combination of immunotherapy with other novel anticancer strategies, such as RT, might improve the therapeutic effects and reduce the side effects of anticancer treatments.

RT has been used routinely in recent decades as one of the primary treatment strategies for more than 50% of cancer patients 3. In general, radiation-induced DNA double-strand breaks and subsequent direct tumor cell death are the major mechanisms by which most solid tumors respond to radiation 4. Radiation could also elicit immune-mediated antitumor responses, triggering the regression of metastatic tumors outside of the local radiation field, which is the so-called abscopal effect 5. However, the overall occurrence of the abscopal effect induced by RT alone is relatively low. This low occurrence may partly be attributed to the insufficiency of RT alone to overcome the immune resistance of metastatic tumors. There has been increasing preclinical and clinical evidence showing that a combination of targeted immunotherapy and RT may lead to improved responses in several tumors, including lung cancer, breast cancer and hepatocellular carcinoma 6. Recently, treatment methods in which PD-L1/PD-1 and/or anti-CTLA-4 ICIs exert systemic effects by restoring antitumor immunity have been combined with RT and demonstrated cytotoxic enhancement via increased immune killing of both irradiated tumor cells and out-of-field tumors 7, 8. Nonetheless, even though combined treatment-induced cases of abscopal effects are now increasingly being reported, they are occurring among only a small proportion of cancer patients in the clinic 9, 10. One of the major reasons could be the immunosuppressive tumor microenvironment (TME) caused by therapy-induced tumor cell death 11. Moreover, specific cell death pathways are activated, leading to either tolerogenic or immunogenic effects on the TME. 12. Therefore, strategies for further improvement of overall response rates are urgently needed, especially targeting the immune response and modulating the TME.

Currently, the use of nanotechnology in RT combined with immunotherapy has shown good potential to overcome these limitations 3. Nanotechnology targets not only cancer cells but also the TME, thereby helping to generate a long-term immune response. Nanomaterials can be an effective delivery system that can encapsulate different kinds of drugs, including both radiosensitizers and ICIs, improving their pharmacokinetics and pharmacodynamics and thus achieving better therapeutic benefits than individual treatment alone 13. Accordingly, some of the approaches have already reached the clinical stage 14, 15. In this review, we focused on the beneficial effects of using nanomaterials combined with RT and immunotherapy against cancers and discussed the in-depth cell death mechanisms. Nanomaterials can regulate the autophagic pathway, which is involved in regulating cancer growth, anticancer effects, the TME, and immune responses. Herein, this review discusses how nanomaterials augment anticancer effects when combined with immunotherapy or RT. The correlation among immunotherapy, RT, nanomaterials, autophagy, and their modulation of cancer cells and TME is discussed. Moreover, the predictive and prognostic biomarkers of this combined treatment are included.

## 2. How does RT modulate the TME and immune response when combined with immunotherapy?

**Figure 1:**
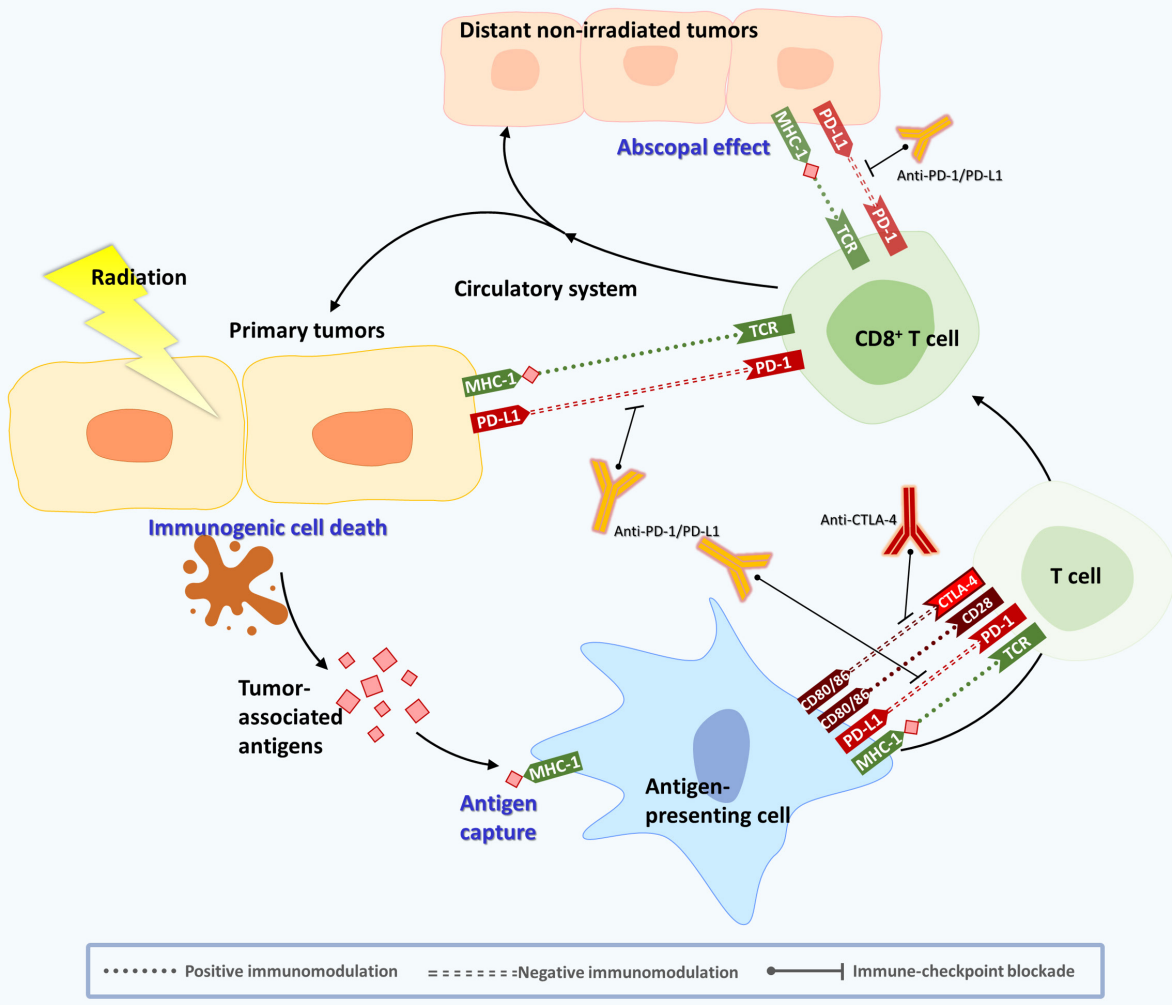
** Schematic diagram of abscopal effects induced by RT combined with ICIs.** RT can activate the human immune system by inducing ICD from cancer cells. After APC uptake, TAAs released from IR-damaged tumor cells move to lymph nodes, and APCs present the antigens to T cells in lymph nodes via the MHC pathway and its costimulatory signals (CD28, CD80, etc.). CD80 and CD86 can activate T cells by binding to CD28. MHC molecules bind to TCRs and activate tumor killing cells. Activated T cells begin to proliferate in lymph nodes and spread through blood and lymphatic vessels. Finally, the activated T cells migrate to the irradiated tumor and any distant tumor lesions to start their antitumor reaction. During this process, immune checkpoints are responsible for maintaining homeostasis of the immune response. CTLA-4 expressed on activated T cells can compete with CD28. PD-1 expressed on NK and T cells can bind to PD-L1 expressed on APCs, which interferes with T-cell-mediated signal transduction. PD-L1 on cancer cells can also send deceptive messages to prevent the immune system from discovering and killing cancer cells. By combining ICIs, such as antibody therapies against CTLA-4 and PD-1/PD-L1, the immunosuppressive effect can be alleviated, and the abscopal effect can be enhanced.

RT is classically known as a strong way to kill cancer cells directly by DNA damage and free radical generation. RT results in cancer cell apoptosis and increases the release of tumor-associated antigens (TAAs) and DAMPs. DAMPs elicit immunological reactions, such as the recruitment of antigen presenting cells (APCs) and subsequent tumor-specific CD8^+^ T cells, to further enhance antitumor responses 16. Antitumor subsets of immune cells, such as CD4^+^ and CD8^+^ T cells, and cytotoxic natural killer T cells are recruited to the surrounding TME and result in immunogenic cancer cell death 17, 18. However, RT also leads to subsequent immunosuppressive effects. DAMPs increase IFN-γ production, which upregulates PD-L1 expression in cytotoxic CD8^+^ T cells, and recruit regulatory T cells via CCR4-binding chemokines 19. Therefore, combination therapy with RT and PD-1/PD-L1 signaling blockade may be a strategy to overcome radioresistance and improve treatment outcomes. Recently, many studies have indicated that the immune system also plays a key role in RT. The interaction between the immune system and RT can be explained by several factors, such as the abscopal effect, TME and immune checkpoint proteins 20. Furthermore, many studies have now developed combination strategies, including immunotherapy or nanotechnology with radiation, hoping to improve treatment effects, enhance immune sensitivity of cancers, and inhibit the growth of metastatic tumors at the same time 21 **(Figure 1).**

### 2.1 Abscopal effect modulation by RT

The abscopal effect noted that RT could also trigger downsizing of metastatic tumors following ionizing radiation treatment of the primary site 22, 23. This magical response has been observed for more than 60 years, but the exact mechanism has not been well clarified before due to its rarity. Fortunately, there is increasing clinical evidence showing an abscopal effect in various tumors, including melanoma, hepatoma, lung cancer, and breast cancer. Researchers have suggested that it may be related to the immune system 24, 25. Interestingly, RT can activate the human immune system by inducing immunogenic cell death (ICD) in cancer cells. ICD leads to the release of proinflammatory cytokines, chemokines, TAAs, and other danger signals to enhance the immunogenicity of tumors. During this process, DAMPs (HMGB1, calreticulin, ATP, etc.) and a series of inflammatory cytokines (TNF-α, interleukins (ILs), interferons, etc.) are secreted. The production of interferon is crucial for RT-induced immune activation 26. Then, antigen-presenting cells (APCs), such as DCs, take up the antigens and move to the lymph nodes to present the antigens to T cells via the major histocompatibility complex (MHC) pathway 27. Activated T cells (especially effector CD8^+^ T cells) will begin to proliferate in lymph nodes and spread through blood and lymphatic vessels. Finally, the activated T cells migrate to the irradiated tumor and to distant tumor lesions to start their antitumor reaction 28, 29. This process can partly explain the regression of distant tumors after local irradiation 5, 30.

### 2.2 Immune checkpoint inhibitors (ICIs) affect the antitumor effects of cancer therapy

However, an overactivated immune response can cause damage to the human body. To avoid this situation, there are some molecules responsible for maintaining homeostasis in the body, called immune checkpoints. For example, CTLA-4 is expressed on regulatory T cells and is upregulated to inhibit T-cell activation, leading to downregulation of the immune response. In addition, immune-inhibitory receptor, PD-1, is expressed on NK and T cells and binds to PD-L1 and PD-L2 expressed on APCs. Accordingly, it interferes with T-cell-mediated signal transduction to limit the immune system's killing effect of cancer cells 5. Several studies have shown that the expression of PD-L1 is upregulated in various types of cancers and is associated with increased tumor aggressiveness and radiation resistance 31. Because CTLA-4 and PD-1/PD-L1 are the main negative immunomodulatory receptors that weaken T-cell activation and the immune response, tumors can use this mechanism to prevent the immune system from discovering and killing cancer cells. Moreover, tumor cells can also attract Treg and myeloid-derived suppressor cells (MDSCs) to prompt T cells to inhibit the immune response, which will lead to rapid growth of tumor cells 32. Many biological factors released by tumor cells in the TME can inhibit immune cell function, leading to immunosuppression 33, 34. The TME consists of different noncellular and cellular components, including tumor cells, fibroblasts or cancer-associated fibroblasts (CAFs), mesenchymal stromal cells (MSCs), pericytes, vasculature, lymphatic networks, myeloid populations, MDSCs, adipocytes, immune cells, and inflammatory cells 35. Noncellular components of the TME are cytokines, chemokines, exosomes, growth factors, inflammatory enzymes, extracellular matrix, and matrix remodeling enzymes 35. Among the TME cells, CAFs are the major cells that secrete growth factors, including hepatocyte growth factor (HGF), epidermal growth factor (EGF), stromal cell-derived Factor 1 (SDF-1), IL-6, exosomes containing miRNAs and others, to mediate tumor growth, monocyte recruitment, immunosuppression, and resistance to therapy 35.

Recent studies have demonstrated that ICIs can inhibit tumor growth by regulating different TME cells 36, 37. For example, CTLA-4 mAb can target and alter Treg cells in the TME and enhance antitumor immunity 38. Another study found that the efficacy of anti-PD-1 therapy depends on DCs, which can secrete IFN-γ, IL-1, and IL-12, thereby establishing a role in promoting anticancer T-cell immunity during checkpoint blockade. Furthermore, the author indicated that activating the noncanonical NF-κB transcription factor pathway amplified IL-12-producing DCs and sensitized tumors to anti-PD-1 treatment, suggesting that this therapeutic strategy might improve responses to immune checkpoint blockade 36 **(Figure 1).** The development of new ICIs and their clinical trials are still ongoing.

### 2.3 Clinical implications for combining RT with immunotherapy: perspectives and challenges

Cytotoxic drugs have been the primary weapon against cancer for a long time for their capacity to kill tumor cells directly. In contrast, immunotherapy treats cancer by targeting the immune system, not the tumor itself 39. It can reshape the TME and enhance the immune system to attack cancer cells in multiple targets and directions. Ablative treatments, such as chemotherapy and RT, are recognized as partners for immunotherapy and may result in a cancer response through a wide variety of mechanisms. These mechanisms include ICD, tumor antigen presentation, tumor cell targeting, or clearance of immunosuppressive cells 40.

Although RT can cause ICD, it also leads to subsequent immunosuppressive effects. A high percentage of PD-1/Tim-3 expression in CD8 T cells was reported in HCC patients after Y90-RE treatment, which shows the exhausted status of immune cells 17. It was also found that low doses of fractionated RT led to PD-L1 upregulation on tumor cells in a variety of syngeneic mouse models of cancer 41. Thus, adding PD-1/PD-L1 signaling blockade to RT may be a strategy to overcome the acquired radioresistance. Compared to monotherapy (RT or ICIs alone), a significant increase in survival was found for RT combined with immunotherapy in animal models. In addition, an abscopal treatment effect was observed to be increased in combined therapy with RT and immunotherapy 42. In the clinic, adding ICIs to chemo-RT in NSCLC patients has achieved encouraging outcomes compared to standard treatment 43. Currently, combination therapy of ICIs and RT has become a promising strategy, while RT can enhance tumor antigen presentation, and ICIs may overcome RT-induced immunosuppressive effects 31. Kim et al. evaluated the combined effect of anti-PD-L1 and RT by using murine HCa-1 cells and found that this combined therapy resulted in the restoration of CD8^+^ T-cell functions, significantly suppressed tumor growth, and improved the survival rate compared to the anti-PD-L1 or RT alone group 44. Another study noted that combining an anti-PD-1 antibody and stereotactic body radiation therapy (SBRT) (30 Gy/3fx) reduced the growth rate of tumors and improved survival in mice injected with Hep-55.1c cells. They also documented that combined therapy could increase the number of CD8^+^ cytotoxic T cells in the tumor and augment the response of the tumor to SBRT 45. In the clinic, Antonia et al. surveyed the impact of adding a maintenance dose of durvalumab (PD-L1 inhibitor) to non-small cell lung cancer (NSCLC) patients after completing chemo RT. The results favored durvalumab in median progression-free survival (16.8 months vs. 5.6 months, P<0.001) compared to placebo. The median time to death or distant metastasis was also longer with durvalumab than with placebo (23.2 months vs. 14.6 months; P<0.001) 46. Another study was designed to evaluate abscopal effects during anti-PD-1 therapy and RT. They included those patients receiving RT within 1 month after the first or last application of nivolumab or pembrolizumab and at least one metastasis outside the irradiation field. Among 24 patients eligible for lesion analysis, 7 patients (3 melanoma, 3 NSCLC, and 1 renal cell carcinoma) developed an abscopal effect (29%). Grimaldi et al. also reported that 52% (11/21) of their patients with advanced melanoma experienced an abscopal effect after combined therapy with ipilimumab and RT. Furthermore, patients with abscopal effects had longer survival than those without immunotherapy (median: 22.4 months vs. 8.3 months) 9. In a report that surveyed 5 patients with unresectable hepatocellular carcinoma who received SBRT followed by anti-PD-1 antibodies. All patients (100%) responded to treatment, with 2 complete responses and 3 partial responses. No tumor progression was noted during the follow-up (median: 14.9 months; range: 8.6-19 months) 47. All these studies suggest that the systemic effects of RT under ICIs could contribute to the development of a broader range of cancer treatments 48.

Nonetheless, the results are not always positive. Kwon et al. showed a disappointing result of ipilimumab and RT combined therapy for metastatic castration-resistant prostate cancer (mCRPC) patients in a phase III clinical trial. There was no difference in the median overall survival of the ipilimumab group compared with the placebo group 49. Similarly, compared to the current standard of sorafenib in advanced HCC, PD-1 inhibitors failed to show superiority 50, 51. This finding means that although the curative effect is remarkable in some patients, it may be ineffective in other patients. Accordingly, there are several questions that need to be addressed, such as how to integrate RT into an immune treatment in patients in the best sequence, accompanied by the best fractionation and dose of RT. How can we identify patients who will benefit from this combined therapy in different cancer types? How do we amplify the systemic antitumor response, such as the abscopal effect, in managing patients with advanced stage disease? In addition, reports of long-term, treatment-related toxicity are still limited. Future trials and research are required to answer these questions and make the use of immune-RT robust. In conclusion, RT combined with ICIs has become a novel treatment to fight against both primary tumors and distant metastases. However, the immune mechanism and optimal strategy of this combination still need further investigation.

## 3. The benefits of nanotechnology in enhancing anticancer effects when combined with immunotherapy and RT

Nanotechnology can improve therapeutic benefits by reducing systemic side effects and increasing drug accumulation inside tumors 52. Nanomaterials can achieve specific tumor targeting via passive targeting utilizing the enhanced permeability and retention (EPR) effect due to the leaky vasculature of cancerous tissues, active targeting (through high-affinity targeting molecules), and triggered release in response to endogenous or exogenous stimuli 47, 53. These properties of nanotechnology can provide numerous advantages by different designations and strategies for combination therapy with RT and immunotherapy that are discussed in the following sections.

### 3.1. Enhanced anticancer effects of RT by nanotechnology

**Figure 2:**
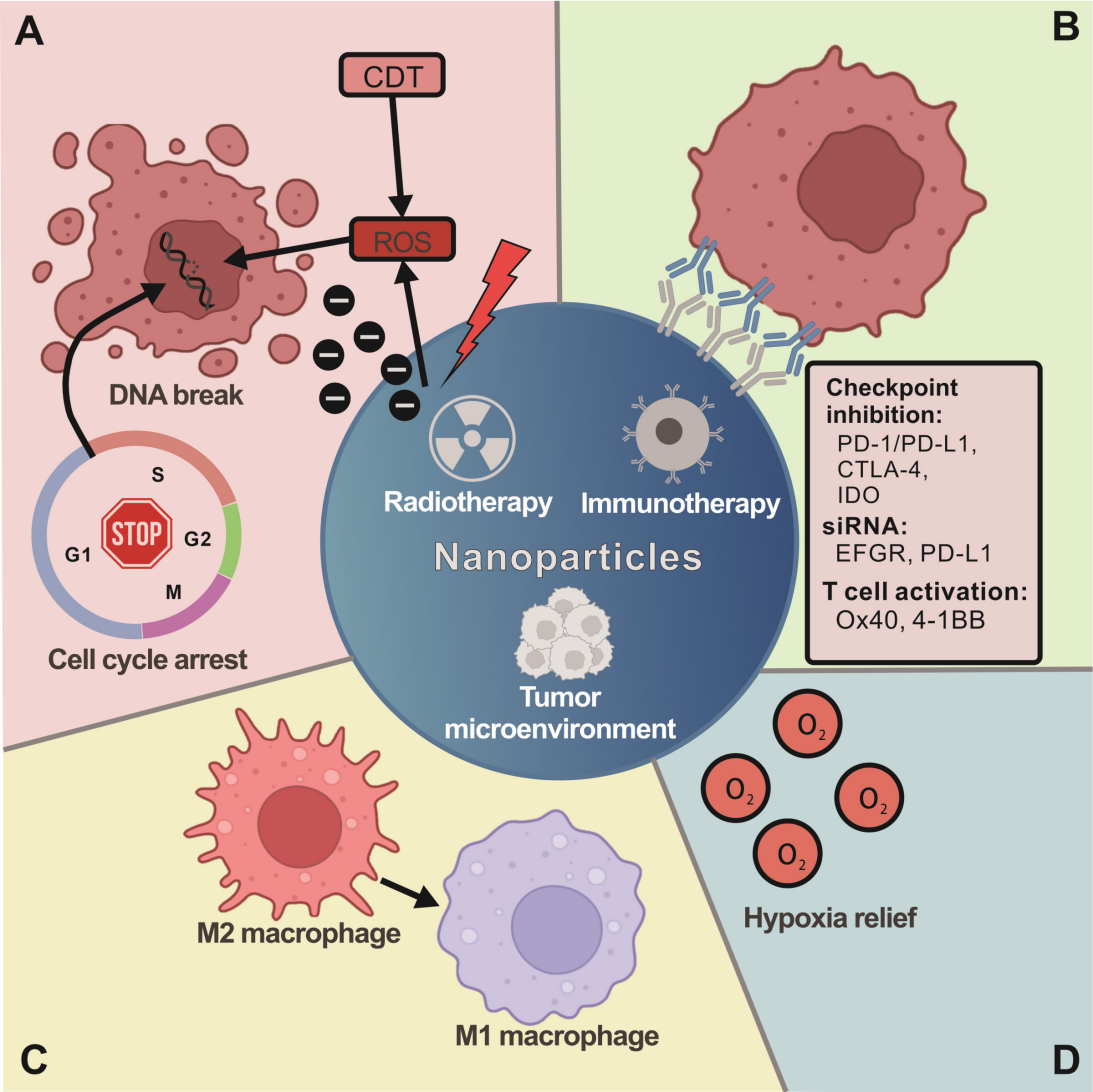
** The benefits of nanotechnology combined with RT and immunotherapy in anticancer therapy.** (A) The properties of nanomaterials can provide numerous advantages by using different strategies for combination therapy with RT and immunotherapy. Nanomaterials combined with RT can enhance radiosensitizing and synergistic effects and increase cellular ROS generation and cell cycle arrest. (B) The nanomaterials can also improve cargo delivery in combination with immunotherapy, conjugation with immune checkpoint inhibition antibodies (PD-/PD-L1, CTLA-4, IDO, Ox40 and 4-1BB) or siRNA. Moreover, nanomaterials can increase antitumor efficacy by targeting the TME. (C) For instance, nanomaterials can alter the TME by mediating the transformation of M1/M2 macrophages. (D) Nanomaterials can also improve the hypoxia status in the TME. In general, these strategies of combining nanomaterials with RT and immunotherapy as anticancer therapy provide novel insight into how to design effective therapies.

Numerous studies have investigated the radiosensitizing and synergistic effects of nanoparticles (NPs) for RT in recent decades, and different types of NPs combined with RT are summarized in **Table 1.** Photoelectrons and Auger electrons generated from irradiated metal-based NPs could contribute to dose enhancement and subsequent radiobiological enhancement 54. The electrons can damage cells directly through interactions with critical targets or indirectly through free radical production. The production of hydroxyl radicals in the radiosensitization processes of metal-based NPs might be attributed to the surface-catalyzed reaction, especially for high-energy charged particles 55. High atomic number (Z) nanomaterials, such as gold NPs (AuNPs), have good biocompatibility and a relatively strong photoelectric absorption coefficient 55 and are suitable for diagnostic and therapeutic applications in cancer RT 56. Other high-Z metals, such as gadolinium 57, platinum 58, and silver 59, have also been reported to enhance RT effects. Metal-based NPs induce cell cycle arrest 60, which can be exploited to increase the radiosensitivity of cancer cells. Roa et al. reported that pretreatment with glucose-capped AuNPs led to the accumulation of cancer cells in the most radiosensitive G2/M phase due to the activation of cyclin-dependent kinases (CDKs) 61. Another synergistic effect of radiosensitization can occur through DNA targeting by NPs 62. Additionally, Kobayashi et al. reported that chloroterpyridine platinum (PtTC) bound to plasmid DNA could enhance the X-ray-induced breaks in DNA mainly mediated by hydroxyl radicals (·OH) in aqueous solution 63. AgNPs showed various biological activities, such as the induction of apoptosis 64, the production of ROS 65, the inhibition of efflux activity by drug-resistant cells 66, and reactivity with glutathione (GSH) molecules 67, which all contribute to the enhanced anticancer effects of RT. Moreover, the specific TME, such as acidity and high H2O2 and GSH levels 68, has been utilized to generate hydroxyl radicals through Fenton-like reactions by transition metal ion catalysis 69, which is also called chemodynamic therapy (CDT) and can trigger ICD in cancer cells that boost immunotherapy 70, 71. Iron ions are mostly used and designed as nanocarriers in iron oxides, zero valent iron (ZVI) forms or metal-organic frameworks (MOFs) to trigger CDT in tumor sites 64, 72-74. Other metal ion-incorporated NPs, such as copper, zinc and manganese, have also been reported to show synergistic effects from a combination of CDT and photo/RT 75, 76 **(Figure 2A)**.

### 3.2 Enhancing immunotherapy with nanotechnology

NPs can improve cargo delivery by targeting tumor cells, increasing drug stability and solubility, and extending circulation half-life 77. Mi et al. developed dual immunotherapy NPs (DINPs) 78 consisting of maleimide-PEG-PLGA NPs with both OX40 and PD-1 antibodies conjugated to the surface. This process allowed for a precise spatiotemporal codelivery of antibodies to simultaneously block T-cell inhibition by PD-1 antibodies and upregulate T-cell activity by OX40 antibodies while significantly increasing the number of CD8^+^ T cells infiltrating the tumor site compared to codelivery of free antibodies 78. A similar strategy was reported by Kosmides et al. using PD-1 and 4-1BB antibodies conjugated to iron-dextran NPs 79. Additionally, NPs can be used as a drug delivery platform and enhance immunotherapy combined with RT. A study used selenium-containing NPs to deliver the chemotherapeutic drug doxorubicin (DOX) to tumor sites by systemic administration, and RT could oxidize selenium NPs and release DOX. This strategy has both synergistic effects and immunomodulatory activity by enhancing NK-cell function 80. Recently, NPs loaded with photosensitizer (PS) and PD-1/PD-L1 antibodies or small molecule ICIs were used to efficiently eradicate local and abscopal tumors, inhibit lung metastasis, and offer long-term immune memory responses to prevent cancer recurrence in a triple-negative breast cancer (TNBC) murine model 81. Another nanoplatform to enhance immunotherapy is membrane-camouflaged nanomaterials. Xiong and his colleagues developed an R837-loaded PLGA nanovaccine coated with a CRT-expressed cancer cell membrane antigen for immunotherapy 82. The DOX-induced ICD provided the whole cancer cell membrane antigen array and exposed CRT to the plasma membrane, which increased the uptake of the NV by communicating an “Eat Me” sign to induce DCs to take up the NV. In another case, Xu and colleagues reported a polymeric multicellular nanoengager (SPNE) coated with fused membranes derived from immunologically engineered tumor cells and DCs 83. Together, nanotechnology shows great potential to improve immune cell therapy for anticancer therapy **(Figure 2B)**.

### 3.3 Nanotechnology-based therapy augments the anticancer effects of RT and immunotherapy

Another approach to anticancer effects is to utilize NPs to improve the immune response post RT, and the studies are summarized in **Table 2.** Erel-Akbaba et al. have shown that radiation followed by the administration of solid lipid NPs conjugated with immunotherapeutic small interfering RNAs (siRNAs) against epidermal growth factor receptor (EGFR) and PD-L1 leads to a significant decrease in glioblastoma growth and improved mouse survival 84. A different type of NP used viral-like particles derived from cowpea mosaic virus (CPMV) as an alternative to siRNAs to elicit an immune response 85. Min et al. used maleimide polyethylene glycol (PEG)-poly(lactic-co-glycolic acid) (PLGA) to form antigen capturing NPs (AC-NPs) to capture neoantigens from dying tumor cells post RT. AC-NPs improved the abscopal response in mice by binding to tumor antigens released following RT and improving their presentation to DCs 86 **(Figure 2C)**. Li et al. also reported that high-Z quantum dots (PbS/CdS QDs) can promote immunogenic RT and abscopal effects against cancer metastasis 87. The QDs promote the immunogenic cell death of cancer cells and trigger the activation of DCs and enhance T-cell-mediated antitumor immunity. The abscopal effect was further boosted when combined with PD-1 antibody, suggesting that nanotechnology can enhance the anticancer therapy of RT combined with immunotherapy.

### 3.4. Nanomaterial-mediated delivery improves antitumor efficacy by targeting the TME

Recent studies have indicated that RT-induced senescence-associated secretory phenotype (SASP) cells release TGF-β and attract immunosuppressive MDSCs, which results in RT resistance and decreased T-cell efficacy 88. Radiation also induces alterations in endothelial cell function and further enhances the aggregation of platelets, forming microthrombi and promoting the adhesion of inflammatory cells within the perivascular space 89. Overall, radiation elicits alterations in the TME through regulation of both tumor and stromal compartments that might activate the immune response, hypoxia, and fibrosis, with the TME leading to recurrence or therapy resistance 35. Accordingly, using NPs to target immunosuppressive cells in the TME offers a promising strategy to eliminate this immunosuppression. Chen et al. showed that local hypoxia could be ameliorated through the use of dual-loaded core-shell PLGA NPs containing water-soluble catalase. These NPs were able to relieve local tumor hypoxia and enhance the effects of RT 90. According to the oxygenation strategy, many catalase-mimicking nanozymes, such as the DNAzyme function of G-quadruplexes and hemin 91, iridium nanocrystals 92, manganese dioxide 93, etc., have been developed to change the hypoxic TME to improve anticancer efficacy. Iron oxide NPs have been reported to inhibit tumor growth by inducing a proinflammatory immune response with M1 macrophage polarization and modulating tumor tissue immunity 94. When combined with a checkpoint inhibitor (anti-PD-L1) and T-cell activators (anti-CD3 and anti-CD28), the iron oxide-based magnetic nanomaterial can repair the immunosuppressive tumor microenvironment (ITM) by reinvigorating tumor-infiltrating lymphocytes 95. Recently, photodynamic therapy (PDT)-mediated ferroptosis-inducing magnetic nanomedicines composed of chlorophyllin-assembled iron oxide clusters further suppressed PD-L1 and IDO-1 expression in bladder cancer cells within CDT. This effect not only mitigated the immune escape ability of cancer cells but also modulated the ITM by enhancing antitumor TAMs and CD8^+^ T-cell infiltration 96.

More recently, zero valent iron (ZVI) NPs have also been revealed as potential immune modulators to augment anticancer immunity by switching protumor M2 macrophages into antitumor M1 macrophages, minimizing the population of regulatory T cells and attenuating PD-L1 expression in cancer cells 97. Thus, iron-based nanomaterials show great potential as adjuvants for cancer immunotherapy. Similar to iron, manganese was recently found to be indispensable for antitumor immunity via activation of the cGAS-STING pathway, which enhances immune cell infiltration and proinflammatory cytokine secretion 98. Thus, manganese-based nanoactivators have also been applied to enhance cancer immunotherapy 99. Moreover, manganese oxide nanomaterials are able to catalyze H^+^/H_2_O_2_ to O_2_ and release Mn^2+^ that can relieve the hypoxic TME, sensitize immunogenic PDT/RT, and promote tumor ablation 93, 100** (Figure 2D)**. Glycopolymer NPs are also reported to improve the capacity to target cancer cells by promoting receptor-mediated endocytosis and endowing multivalent effects for activating immune signaling in immune cells 101. Recently, Huang et al. developed a unique polymerization method to produce Au NPs coated with polyaniline containing galactoside to achieve high galactose moiety exposure that enhanced the internalization of AuNPs and induced over 90% M1 repolarization from M2 polarized macrophages 102 and reversed the ITM from cold tumors to hot tumors in lung cancer when combined with anti-PD-1 treatment 103. Collectively, modulation of the TME is one of the benefits of combination with nanotechnology and RT or ICIs to augment their anticancer effects.

### 3.5 The advantages, challenges and future expectations for nanotechnology combined with RT and immunotherapy

Although the advantages of nanotechnology in drug delivery were discussed in a previous report 104, small nanoparticles (< 50 nm), which are the optimized size for endocytosis 105, might have the disadvantage of a low drug loading concentration. In a nanocarrier therapeutic system, undesired burst release occurs in the physiological environment 106, which requires the biochemical design of a passivation layer on the particle surface to restrict drug leakage. The other challenge is the limitation of complete release (70-90%) either by light, pH, enzyme reaction, or magnetic waves in the controlled release nanocarrier system 107, 108 and thus would suffer from an insufficient drug dose in the lesion with practical clinical trial applications. Most importantly, the surface of RT-activated nanoparticles can simply modify the biomolecules 109 to improve the water dispersity and increase the targeting rate without concern for the retention of the chemical drugs on the surface or in the core area structures by a complicated layer-by-layer coating process 106.

Indeed, nanotechnology can also greatly help improve RT and immunotherapy through other routes. First, a strong photoelectric absorption coefficient can generate photoelectrons and Auger electrons from irradiation to enhance RT-induced ICD and RT efficiency by high-Z materials. Second, the CDT-catalyzed reaction from transition metals in a high catalytic environment (such as an acidic environment and high H_2_O_2_ and GSH concentrations in the TME) can further weaken cancer cells to achieve radiosensitization processes and induce ICD to boost immunotherapy. Third, delivering antitumor drugs, radiosensitizers, oxygen, ICIs, and genetic regulatory RNAs or providing antigen capturing/presenting function can reverse immunosuppressive TME and enhance RT and immunotherapy by membrane camouflage or engineering-designed polymeric NPs. Finally, immunomodulation by TAM repolarization from a protumor to an antitumor phenotype induced antitumor immunity by iron-based materials and glycopolymer-composed NPs **(Figure 2)**.

Regarding the RT-responsive nanoparticles activated upon radioactive source stimulation 110-112, the killing efficiency of cancer cells was generally dependent on the radiation energy when compared with the controlled release of the drug carrier system. It can achieve localized treatment by controlling the irradiation area and avoiding adverse effects by reducing unexpected drug release in the nonlesion region. This side effect is avoidable when patients receive < 2 Gy dose per fraction according to a clinical report 113. While the use of nanoparticles as RT sensitizers can largely reduce the high RT dose from 20-60 Gy in clinical settings 114 to below 10 Gy, the threshold causes significant vascular damage 115. Some cases can be reduced to 2 Gy or below, such as Au at 4-6 Gy 116, MnO2 at 3 Gy 117, BSA-decorated gold nanoclusters at 6 Gy 118, Hf, a first-in-class radioenhancer known as NBTXR3 (Nanobiotix), at 2-5 Gy 119-121, Bi at 9 Gy 122, Bi2O3 NPs at 6 Gy 123, 124, Bi@BSA at 4 Gy 125, FePt nanoparticles below 8 Gy 126, Gd-polysiloxane NPs (AGuIX®) at 2-7 Gy 127, 128, and PbS/CdS QDs at 8 Gy 87. Indeed, controlling the energy dose to regulate cancer eradication and immune activation is an interesting issue. It is worth mentioning that a low radiation dose of 2 Gy can promote inducible nitric oxide synthase expression by tumor-associated macrophages, suggesting that a proimmunogenic environment can be induced by radiation treatment at low doses 129. Although most of the RT doses reported in combination with NPs were under a low energy source, suggesting that NPs can reduce the threshold of RT dose to induce an abscopal effect 109, 130, the systemic investigation of different RT doses and fractions with nanomaterials is still lacking.

As discussed above, nanocomposites with high-Z elements (such as Pt, FePt, Hf, Au, Bi, and Gd) play a vital role in possessing a strong photoelectric absorption coefficient and can generate photoelectrons and Auger electrons from irradiation to enhance RT-induced ICD and RT efficiency. The transition metals (i.e., Mn, Fe, and Cu) showed enzyme-like reactions 131 by the redox reaction in the presence of H2O2, which is promising for evoking CDT to change the TME. The cooperation of both RT and CDT 132 is promising for regulating innate immunity (i.e., M1 macrophage polarization by inflammation 133 and boosting adaptive immunity by the potential biochemical mechanisms of activated DCs and T cells through the RT-induced ICD route of cancer cells, such as antigen release, ATP release, and CRT translocation to the plasma membrane 134.

Despite the advantages of RT-based NPs, additional delivery of 0.1 Gy-induced drug release 110, immune checkpoint blockade 135, iron-based materials 96, NO release 136, and immune-responsive camouflaged nanomaterials 82 can enhance the immunotherapy effect. However, there are some clinical issues that urgently need to be addressed. For example, the effect of nanomaterial-induced autophagy on cancer immunomodulation has rarely been discussed. Another issue is that most studies have focused on RT-induced ICD in cancer cells and subsequent immune activation, and fewer studies have discussed how nanomaterials affect immune cells to reverse the immunosuppressive TME and then enhance anticancer effects. How to synergize the anticancer ability and immune activation ability by one nanomaterial design or by orchestrating multimodal nanosystems remains to be solved and will greatly improve the outcomes of RT and immunotherapy combinations.

## 4. The pivotal role of autophagy in the anticancer mechanism of nanotechnology combined with immunotherapy and RT

As published previously, anticancer therapies induce various types of cell death. Different types of cell death mechanisms may contribute to enhancing or inhibiting the immune reaction in the TME. In this review, we discuss the immune response that could be regulated by different cell death mechanisms and suggest that autophagy could be an important modulator of anticancer effects when using nanotechnology combined with immunotherapy and RT.

### 4.1Cell death mechanisms in regulating the immune response

Anticancer therapy could induce different cell death types, including apoptosis, autophagy, necrosis, ferroptosis, pyroptosis, and necroptosis **(Figure 4A)**. The different immune responses are activated in response to cell death types, thereafter affecting therapeutic effects. For instance, apoptosis and autophagy do not induce additional inflammatory responses 137. The other two ICDs are necroptosis and pyroptosis, which are prone to release inflammatory mediators and alter the inflammatory state of the TME 138. In contrast, inhibition of the components of necroptosis, including RIPK1, RIPK3, and MLKL1, showed reduced tumorigenesis or metastasis in some settings. In addition, pyroptosis is triggered by the binding of PAMPs and DAMPs with pattern recognition receptors (PRRs) and inflammasomes. Inflammasome complex activation induces the cleavage and activation of caspase-1, which promotes pyroptosis via the cleavage of the inflammatory cytokines IL-1β and IL-18 into their mature forms and then cleaves GSDMD, leading to a type of cell death called pyroptosis 139. Loss of GSDMD expression could lead to immune evasion and resistance to immunotherapy 140. In contrast, one previous study indicated that GSDMD and GSDMD-N were upregulated in HCC because GSDMD promoted PD-L1 expression by the Ca^2+^/histone deacetylase (HDAC)/signal transducer and activator of transcription 1 (STAT1)-induced transactivation pathway 141. This study indicated the oncogenic role of GSDMD in HCC 141. Taken together, different types of ICD could exist in the tumor simultaneously, and the crosstalk among ICDs could influence anticancer effects after treatment 142. Further mechanisms of how ICDs talk with each other and how they interfere with the therapeutic efficacy of anticancer treatment still await more investigation. Autophagy is one of the different types of cell death. Autophagy can also contribute to cell survival. This contribution is especially true in cancer cells. The dual roles of autophagy are a unique feature of cancer therapy.

### 4.2 The pivotal roles of autophagy in cancer therapy

**Figure 3:**
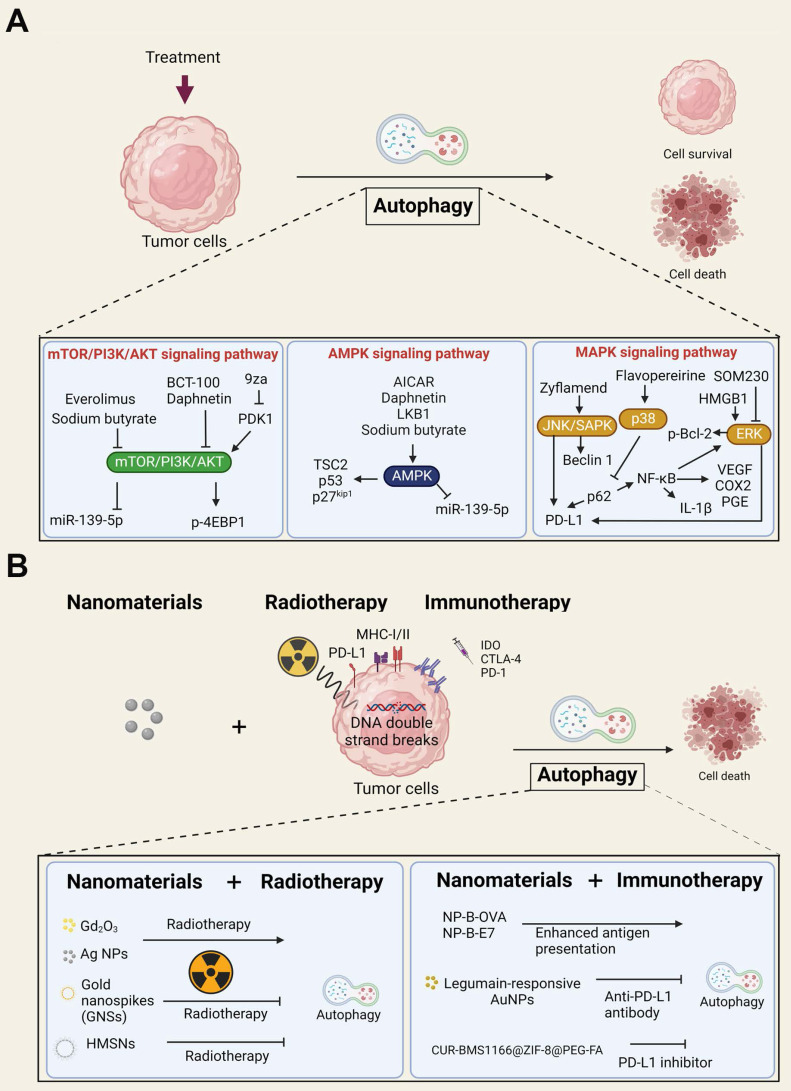
** The autophagy signaling pathway is linked to cancer subjected to various treatments. (A)** An overview and schematic depiction of autophagy-related signaling pathways in cancer cells. The mTOR/PI3K/Akt, AMPK, and MAPK signaling pathways are the three major autophagy-related pathways in tumor cells. Abnormal mTOR/PI3K/Akt signaling in cancer cells is related to tumor growth, cancer cell survival and cytoskeletal movement. AMPK is important in regulating energy homeostasis. By supporting energy metabolic processes, AMPK supports cancer cell proliferation. MAPK signaling pathways can be classified into three main families: the ERK, JNK/SAPK and p38 kinase families. **(B)** An overview and schematic depiction of nanomaterials and their therapeutic implications for RT or immunotherapy. These nanomaterials combined with RT or immunotherapy induced autophagy and caused the death of tumor cells.

Autophagy (microautophagy, macroautophagy, and chaperone-mediated autophagy) plays an important role in many eukaryotes, and autophagy-related genes (Atgs) are highly conserved. The autophagy processes include phagophore assembly (initiation, nucleation, and expansion), autophagosome formation (maturation), and autolysosome degradation 143. Autophagy plays dual roles in promoting or inhibiting tumorigenesis 144. For instance, autophagy could play a critical role in optimal immune function through the regulation of danger signaling in neoplastic cells under immunogenic chemotherapy and/or RT 145. In contrast, nanomaterial-induced autophagy may play an important role in enhancing or inhibiting anticancer therapy 150. Accordingly, understanding the molecular regulatory pathways and the crosstalk between cell death, such as autophagy, and anticancer therapy may help us achieve maximal therapeutic efficacy. In this section, we briefly discuss the three major autophagy pathways involved in anticancer therapies **(Figure 3A)**.

The first pathway is mTOR/PI3K/Akt signaling, which is dysfunctional in many cancers and is related to cancer cell growth, cancer cell survival, cytoskeletal movement, and resistance to cancer therapy 146. The mTOR downstream regulator p-4EBP1 is a critical prognostic marker in ovarian cancer 147. It has been reported that PEGylated recombinant human arginase 1 (BCT-100) induces autophagy and apoptosis by regulating the ROS/AKT/mTOR pathway in bladder cancer cells 148. Similarly, sodium butyrate activated autophagy via the AMPK/mTOR pathway and ROS-mediated apoptosis and inhibited migration by the miR-139-5p/Bmi-1 signaling pathway in bladder cancer cells 149. In contrast, daphnetin triggered ROS-induced cell death and induced cytoprotective autophagy by modulating the AMPK/Akt/mTOR pathway in ovarian cancer 150. Accordingly, clinical trials have approved many PI3K/Akt/mTOR inhibitors for cancer treatment, such as everolimus for mTOR inhibition 151, 152. The second pathway is AMPK, which is an important factor in the response and maintenance of energy homeostasis. AMPK also acts on downstream factors, such as TSC2 and p53, and mediates cancer formation and development 153. LKB1 is the upstream tumor suppressor of AMPK, indicating that AMPK plays a central role in tumor inhibition 154. Presently, AICAR, an AMPK agonist, promotes growth arrest, autophagy, and has anti-proliferation properties and inhibits the migration and invasion of triple-negative breast cancer *in vitro* 155 and in clinical trials 156.

The third important mechanism is the MAPK signaling pathway, including the extracellular signal-regulated kinase (ERK), C-Jun N-terminal kinase/stress-activated protein kinase (JNK/SAPK), and p38 kinase signaling pathways 157. A previous study demonstrated that JNK had a significant oncolytic effect, and autophagy inducers acting on the JNK/Beclin-1 axis may enhance this oncolytic effect 158. Zyflamend, an herbal extract with anti-inflammatory properties, induces pancreatic cancer apoptosis by inducing autophagy and JNK signaling 159. In contrast to the anticancer effect of autophagy, the ERK inhibitor pasireotide (SOM230) has been demonstrated to target pituitary adenoma tumorigenesis and inhibit autophagy 160. SOM230 is currently used in clinical trials 142, 161. Consistently, immune signals such as HMGB1 are widely recognized to induce autophagy 162 and promote phosphorylation of Bcl-2 and dissociation of Beclin1-Bcl-2 through the ERK pathway 163. As a result, the three major autophagy pathways play vital roles in the dual function of autophagy in anticancer therapies and may be applied in clinical settings when the autophagy pathway is manipulated in an appropriate manner.

### 4.3. The dual roles of autophagy in RT, immunotherapy, and nanotechnology

Research in our laboratory and by other groups over the last 10 years has revealed nanomaterials to be strong autophagy inducers with the ability to elevate autophagy to very high levels. One of our previous works showed that autophagy activated by silver NPs failed to trigger the lysosomal degradation pathway, leading to defective autophagic flux or autophagy dysfunction 164. In addition, autophagy dysfunction could trigger inflammasome activation and exosome release, thereby enhancing the immune response 165-167. In most cases, autophagy induced by nanomaterials promotes cell death. However, certain nanomaterials are also able to elicit prosurvival autophagy 168. At present, whether NP-induced autophagy has the potential to increase the effectiveness of RT and immunogenic chemotherapy is still unknown. A study showed that autophagy induced by irradiation could promote cell death of human colorectal cancer cells 169. Similarly, irradiation induced cell damage in Atg7-deficient neural stem cells 170. Moreover, studies have shown that autophagy-related microRNAs play an essential role in improving RT in renal and bladder cancer 171.

Knowing the relationship between autophagy and tumor immunological tolerance can be a crucial event for improving tumor immunotherapy. Induction of autophagy can decrease the inflammation-activated expression of IDO to inhibit tumor development in the cervical cancer cell lines HeLa and SiHa and promote the induction of phagocytosis in macrophages 172. Autophagy activators are also available to suppress primary resistance to CTLA-4 blockade by decreasing MAGE-A protein levels and improving anti-CTLA-4 curative effects 173. Similarly, combined RT, mTOR and PD-1 inhibitors could enhance radiosensitivity in cervical cancer by promoting autophagy 174. Consistently, Tsai et al. indicated that autophagy blockade by inhibitors increased PD-L1 expression through the ERK and JNK signaling pathways in bladder cancer cells 175. In contrast, other studies have shown that PD-L1 can inhibit autophagy by activating mTORC1 signaling and inhibiting mTORC2 signaling 176. Notably, a sandwich therapy strategy of RT combined with PD-L1 blockade and autophagy inhibition was reported to enhance the activation of CD8^+^ T cells and improve the curative effect in both irradiated primary tumors and nonirradiated abscopal tumors 177.

The above studies strongly support the dual role of autophagy in immunotherapy. Therefore, if the induced autophagy is a pro-death pathway, it should be maximized. Otherwise, it should be inhibited to enhance the anticancer effects. Nanomaterials usually induce pro-death autophagy, which serves as a significant mechanism for enhancing cancer therapy effects 168. In this context, we expected that autophagy is the key mechanism by which nanomaterials augment anticancer effects when combined with immunotherapy and RT. Indeed, a study demonstrated that ultrasmall gadolinium oxide (Gd_2_O_3_) nanocrystals induced autophagy by sensitizing NSCLC cells to irradiation 178. Studies have shown that silver NPs have antiproliferative, proapoptotic, and autophagic effects to enhance the effectiveness of RT in a glioma model 179. Furthermore, Ge et al. designed a pH-sensitive autophagy-controlling nanocarrier to boost the immunotherapeutic response of PD-1/PD-L1 blockade by activating ICD via autophagic cell death and sensitizing antitumor T-cell immunity in the treatment of osteosarcoma 180. A previous study also indicated that autophagy stimulation by Beclin-1-loaded polymeric NPs significantly diminishes breast cancer progression. Polymeric NPs are designed to protect against Beclin-1 degradation by enzymes, thereby preventing nonspecific biodegradation of nanomaterials 181. Interestingly, current studies indicated that breast cancer is genetically linked to autophagy impairment and Beclin-1 is monoallelically deleted in nearly 50% of sporadic breast carcinomas 182. Autophagy defects in TNBC cells also inhibit T-cell tumor-killing activity, indicating that defective autophagy plays an important role in immunosuppression 183. As a result, autophagy induction by nanomaterials seems to be an attractive therapeutic strategy against tumors with defective autophagy mechanisms. On the other hand, AuNPs combined with an anti-PD-L1 antibody improved glioma treatment by autophagy inhibition 184. The gold nanospikes (GNSs) of radiosensitizers with different modifications demonstrated that autophagy plays a protective role for cancer cells during RT, and the inhibition of prosurvival autophagy increased radiation-facilitated cell death 185. Accordingly, previous studies demonstrated that nanomaterials augment the anticancer effects when combined with RT or immunotherapy through autophagy, as shown in Figure 3B. Taken together, we strongly propose that the application of nanomaterials that could induce pro-death autophagy may be a good strategy to potentially enhance the sensitivity to immunotherapy or RT of such cancers with defective autophagy. Nevertheless, nanomaterials seem to be crucial since they have dual roles in regulating cell death or survival, modulating the TME, and determining the sensitivity to therapy. The role of autophagy should be carefully investigated when clinically applied to anticancer therapy by nanomaterials combined with immunotherapy or RT.

## 5. Predictive and prognostic biomarkers of RT combined with immunotherapy

**Figure 4:**
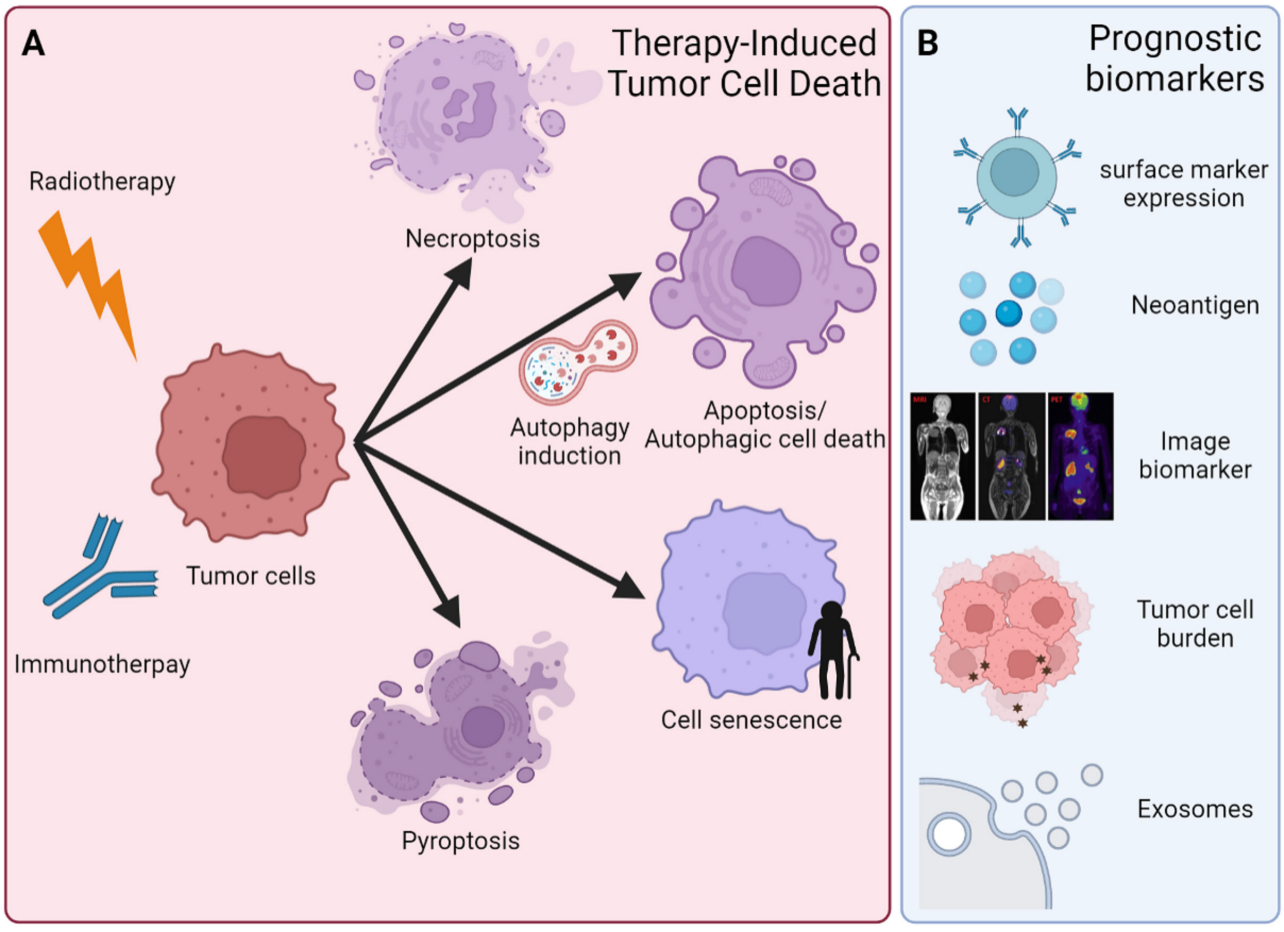
** Schematic depiction of different cell fates and potential biomarkers of RT combined with immunotherapy. (A)** An overview of different types of cell death and cell fate after RT and immunotherapy, including necroptosis, apoptosis, pyroptosis, autophagic cell death (ACD) and cellular senescence. In addition, there is a growing demand for predictive and prognostic biomarkers of RT and immunotherapy. **(B)** Biomarkers are currently validated for clinical practice and research, including surface marker (PD-1, CTLA-4, etc.) expression in tumor cells and immune cells, neoantigen expression, noninvasive image-based technologies (SPE-CT, PET-CT and MRI), tumor mutation burden (TMB), and exosomes.

To date, there is a growing demand to develop predictive and prognostic biomarkers of RT and immunotherapy. Dynamic biomarkers are also used to investigate how treatments may influence key transitions and potentially identify responders and nonresponders in real time, sparing patients toxicities from ineffective treatments and potentially guiding therapy decisions to more effective approaches, especially combined with novel anticancer strategies such as nanotechnology in the future **(Figure 4B)**. Several potential biomarkers are discussed in the following section.

### 5.1 Surface markers

The level of PD-L1 within tumors has been suggested to be a promising prognostic biomarker in patients undergoing RT, but the role of PD-L1 as a predictive biomarker still needs further investigation 186. PD-L1 expression after 12 Gy carbon-ion irradiation showed better progression-free survival in human uterine cervical adenosquamous carcinoma 186. Hematological malignancies such as Hodgkin lymphoma also show PD-L1/PD-1 upregulation after chemotherapy or RT 187. However, the utility of this biomarker is still unclear due to multiple unresolved problems, such as variable detection antibodies, differing IHC cutoff values, tissue preparation, processing variabilities, primary versus metastatic biopsies, oncogenic versus induced PD-L1 expression, and staining of tumor versus immune cells 186.

CTLA-4 can be detected in normal human serum, and higher levels of soluble CTLA-4 in serum have been observed in many types of cancers 188. Pistill et al. demonstrated that higher serum levels of sCTLA-4 (>200 pg/ml) at baseline had both better ORR and OS than lower sCTLA-4 serum levels (<200 pg/ml) in melanoma patients treated with ipilimumab, suggesting that serum sCTLA-4 could be a biomarker for better response to ipilimumab 189.

### 5.2 Genomic Mutations

Neoantigens are small peptide epitopes from tumor-specific mutations. Mutations in the protein-coding regions of DNA generate truncated proteins termed ''neoantigens.'' Neoantigens result in a higher degree of foreignness to cells, which helps immune cells readily target and eliminate tumor cells 190. Neoantigens are tumor-specific and patient specific. The recognition of neoantigens may be an important driver of the anti-immune responses to T-cell targeting therapy, including ICI 191. However, there are still some problems at the experimental stage. For example, only approximately 1% of tumor mutations generate a neoantigen with enough affinity for MHC to prime T cells to respond. Therefore, determining how to define a high-quality neoantigen that can trigger robust immune responses represents a significant challenge.

Other potential biomarkers include the total number of genetic mutations in cells, called the tumor mutation burden (TMB). TMB, defined as the total number of somatic mutations per coding area of a tumor genome, is an emerging clinical biomarker associated with the response to ICI therapy 192. TMB has been shown to vary markedly among tumor types and among patients within tumor types. A higher TMB is commonly observed in cancers associated with mutagens, such as ultraviolet light exposure in melanoma and smoking in NSCLC 192. An association between TMB and improved efficacy of immunotherapies has been reported for several tumor types, suggesting that TMB-H may be a potential biomarker associated with the response to these treatments 193.

### 5.3 Molecular imaging biomarkers

In recent years, noninvasive and clinically useful image-based techniques such as SPE-CT, PET-CT, and MRI have been developed to evaluate the immune response to RT and/or immunotherapy 194. The development of advanced imaging techniques may eliminate unsuitable, painful biopsies and help guide clinical decisions. A European cohort study tested 208 classical Hodgkin's lymphoma patient samples that were treated with two ABVD (doxorubicin (Adriamycin), bleomycin, vinblastine, and dacarbazine) courses and then analyzed the baseline staging and interim restaging with FDG-PET (fluorodeoxyglucose) scans. They found that an early-interim FDG-PET scan after two ABVD chemotherapy courses was the only parameter that could predict both progression-free survival and overall survival 195. In PET-2-negative patients, the expression of CD68 and PD-1 in microenvironmental cells and STAT1 negativity in Hodgkin Reed Sternberg cells identified a subset of PET-2-negative patients with significantly lower 3-year progression-free survival than the rest of the PET-2-negative population 195. This finding indicated a combined role of biomarkers and interim PET scans for the prediction of treatment outcomes and provided new insight into developing immune cancer biomarkers.

### 5.4 Exosomes

Exosomes are a subtype of extracellular vesicles (EVs) between 50 and 130 nm in diameter that are enriched in a set of molecular markers with an endosomal source 166. Exosomes contain various types of molecules, including proteins, RNA, and DNA. Exosomes isolated from the plasma of cancer patients contain various immune-related proteins, including PD-1, PDL1, and CTLA-4, with PD-L1 in exosomes showing a suppressive effect on T-cell activities by signaling via PD-1 196. However, opposite results were observed in the association of exosomal PD-L1 mRNA expression with the response to anti-PD-1 antibodies in patients with melanoma or NSCLC 197. For another example, Tucci et al. demonstrated that increased exosomal PD-1 and CD28 levels in T cells were significantly associated with longer progression-free survival and overall survival, while increased exosomal CD80 and CD86 in dendritic cells correlated with longer progression-free survival. Such exosomal proteins may reflect potential T-cell/dendritic cell activities and thus lead to predictions of ICI response 198.

Overall, there is still much work to be done to identify and develop novel biomarkers for RT and immunotherapy in clinical applications and combined with other anticancer therapies in the future. Although numerous predictive biomarkers for tumor response and ICI treatment have been identified, there are no single biomarkers that can predict tumor treatment response. Therefore, multiple biomarkers should be considered in determining the therapeutic effects of anticancer treatments.

## 6. Conclusions and Perspectives

In the era of cancer therapy, incorporating NPs with RT or immunotherapy holds much promise for expanding therapeutic options for patients. Going forward, it would be important to verify the therapeutic benefits of mechanistic studies of cell death mechanisms and regulation of the TME. Activation of multiple cell death pathways highlights a close relationship between apoptosis, autophagy, necroptosis, and pyroptosis for immunotherapy in combination with NPs, RT, or immunotherapy 199. Among these pathways, autophagy seems to be crucial since it has a dual role in regulating cell death or survival, modulating the TME, and determining the sensitivity to therapy. Notably, the autophagy response might be affected by different treatment strategies in tumor cells, including NPs. Interestingly, many NPs might regulate autophagy by themselves or through the incorporated drugs. While there are still many issues to be addressed to further improve the efficacy of NPs combined with RT or immunotherapy and many mechanisms, including autophagy, regarding the regulation of cell death and TME remain to be further investigated, there is no doubt that NPs could augment the anticancer effects when combined with RT and/or immunotherapy, which may open a new era for clinical oncology. Therefore, it is also necessary to establish models of biomarkers that are applicable for monitoring treatment efficacy when using NPs combined with RT or immunotherapy.

## Figures and Tables

**Table 1:** Summary of studies on nanoparticles combined with RT

Nanoparticle type	Radiation source	Outcome	Reference
Bismuth, gold and platinum NPs	X-ray	Nanoparticles contribute to dose enhancement and subsequent radiobiological enhancement in endothelial cells	Hossain, M., & Su, M. (2012) 54
Citrate-capped AuNPs	X-ray	Radiation enhancement effects in HeLa cells	Liu, Yan, et al. (2015) 55
FePt NPs	X-ray	Radiation enhancement effects in SR3A cell	Tsai, Tsung-Lin, et al. (2021) 58
Silver and gold NPs	6 Gy IR	Radio-sensitization effect in C6 glioma cells	Xu, Ruizhi, et al. (2009) 59
Gold NPs	X-ray	G2/M phase arrest in DU-145 cells	Roa, Wilson, et al. (2009) 61
nMOFs	X-ray	Radiation enhancement in 4T1 cells	Ni, Kaiyuan, et al. (2018) 73

**Table 2:** Summary of studies on nanoparticles combined with immunotherapy and RT

Nanoparticle type	Radiation source	Outcome	Reference
Solid lipid nanoparticle +siRNA-EGFR & PD-L1	5 Gy IR	Significant decrease in glioblastoma growth and improved mouse survival in glioblastoma mouse model.	Erel-Akbaba, Gulsah, et al. (2019) 84
viral-like particles (CPMV)	10 Gy IR	Elicit an immune response (cold tumor to hot tumor) in ovarian cancer cell line ID8-Defb29/Vegf-A.	Patel, Ravi, et al. (2018) 85
Antigen-capturing nanoparticles (AC-NPs)	8 Gy X-ray	AC-NPs combine RT improved the abscopal response in B16-F10 and 4T1 cell lines mouse model.	Min, Yuanzeng, et al. (2017) 86
Anti-4-1BB & anti-PD-L1 immunoswitch particles	-	Immunoswitch NPs increase tumor-infiltrating CD8^+^ T cells activity in *in vivo* B16-SIY model.	Kosmides, Alyssa K., et al. (2017) 79
Selenium-containing nanoparticles + doxorubicin	γ-ray	Radiation could oxidize diselenide-containing NPs to seleninic acid, which have both synergistic antitumor effect and immunomodulatory activity through enhancing NK cells function.	Gao, Shiqian, et al. (2020) 80
BMS-202 nanoparticles	Photodynamic Therapy	BMS-202 nanoparticles could efficiently eradicate local and abscopal tumors, inhibit lung metastasis, and offer long-term immune memory responses to prevent cancer recurrence in a triple-negative breast cancer (TNBC) murine model (4T1 cell lines mouse model).	Zhang, Rui, et al. (2019) 81
High-Z quantum dots (PbS/CdS QDs)	X-ray	High-Z quantum dots (PbS/CdS QDs) promote immunogenic RT and abscopal effects to against cancer metastasis in 4T1 tumor-bearing mice.	LI, Hao, et al. (2021) 87
PLGA-R837@Cat NPs	X-ray	NPs were able to relieve local tumor hypoxia, enhancing RT.	Chen, Qian, et al. 90
Fe_3_O_4_@Chl/Fe CNPs	Photodynamic Therapy	Photodynamic therapy (PDT)-mediated ferroptosis inducing Fe_3_O_4_@Chl/Fe CNPs clusters could further suppress the PD-L1 and IDO-1 expression in bladder cancer cells.	Chin, Yu-Cheng, et al. (2022) 96

## Abbreviations

RT: RT
ICIs: Immune checkpoint inhibitors
TME: Tumor microenvironment
PD-1: Programmed death 1
PD-L1: Programmed death ligand 1
CTLA-4: Cytotoxic T-lymphocyte associated protein 4
DAMPs: Damage-associated molecular patterns
RT: RT
ICD: Immunogenic cell death
TAAs: Tumor-associated antigens
DC: Dendritic cell
APCs: Antigen-presenting cells
TLRs: Toll-like receptors
MHC: Major histocompatibility complex
TCRs: T-cell receptors
Tregs: Regulatory T cells
MDSCs: Myeloid-derived suppressor cells
NSCLC: Non-small cell lung cancer
mCRPC: Metastatic castration-resistant prostate cancer
HCC: Hepatocellular carcinoma
SBRT: Stereotactic body radiation therapy
EPR: Enhanced permeability and retention
AuNPs: Gold nanoparticles
CDKs: Cyclin-dependent kinases
EGFR: Epidermal growth factor receptor
CPMV: Cowpea mosaic virus
PLGA: Poly(lactic-co-glycolic acid)
AC-NPs: Antigen capturing NPs
PDT: Photodynamic therapy
PS: Photosensitizer
IDO: Indoleamine 2,3-dioxygenase
ITM: Immunosuppressive tumor microenvironment
ICDs: Immune checkpoint blockades
UCNPs: Upconversion nanoparticles
TNBC: Triple-negative breast cancer
MSCs: Mesenchymal stromal cells
CAFs: Cancer-associated fibroblasts
HGF: Hepatocyte growth factor
EGF: Epidermal growth factor
SASP: Senescence-associated secretory phenotype
ADCD: Autophagy-dependent cell death
Atgs: Autophagy-related genes
ERK: Extracellular signal-regulated kinase
JNK/SAPK: C-Jun N-terminal kinase/stress-activated protein kinase
AgNPs: Silver nanoparticles
ASN: Autophagy cascade amplification nanoparticle
NPs: nanoparticles
